# Leisure time activities as mediating variables in functional disability progression: An application of parallel latent growth curve modeling

**DOI:** 10.1371/journal.pone.0203757

**Published:** 2018-10-03

**Authors:** Ya-Mei Chen, Yu-Kang Tu, Hsiao-Wei Yu, Tzu-Ying Chiu, Tung-Liang Chiang, Duan-Rung Chen, Ray-E Chang

**Affiliations:** 1 Institute of Health Policy and Management, College of Public Health, National Taiwan University, Taipei, Taiwan, R.O.C; 2 Institute of Epidemiology and Preventive Medicine, College of Public Health, National Taiwan University, Xu-Zhou Road, Taipei, Taiwan, R.O.C; 3 Department of Gerontological Care and Management, Chang Gung University of Science and Technology, Kwei-shan,Taoyuan,Taiwan, R.O.C; 4 Graduate Institute of Long-term Care, Tzu Chi University of Science and Technology, Hualien City, Taiwan, R.O.C; 5 Institute of Health Behavior and Community Sciences, College of Public Health, National Taiwan University, Taipei, Taiwan, R.O.C; West Chester University of Pennsylvania, UNITED STATES

## Abstract

**Objectives:**

The aims of this study were to investigate (1) whether and (2) the extent to which Taiwanese older adults’ leisure time activity (LTA) trajectories mediated the potential association between their sociodemographic factors and their functional disability trajectories.

**Methods:**

Longitudinal data from four waves of the Taiwan Longitudinal Study on Aging (TLSA), collected between 1996 and 2007, were used for analysis (*N* = 3,429). Parallel-process latent growth curve modeling was adopted to evaluate the process by which LTA mediated between sociodemographic factors (age, gender, education, self-rated health, comorbidities, and depression) and the outcome process of functional disabilities.

**Results:**

When mediated by baseline level of LTA, five sociodemographic factors—age, gender, education level, self-rated health, and number of comorbidities—had significant and negative mediating effects on baseline or change in functional disability, thus improving disability outcomes. However, four of the sociodemographic factors (age, education level, and number of comorbidities), when mediated through the rate of change in LTA, were found to have significant and positive mediating effects, which increased disability levels. The proportion of effects mediated by the LTA trajectory ranged from 0% to 194%.

**Discussion:**

The large proportion of effects mediated through the LTA process underlines the importance of LTA to public health policy and health programs for older adults. The study’s findings shed light on how to better target populations of older adults to promote an active lifestyle and achieve more successful aging in late life in Asian countries.

## Introduction

Engagement with life and decrease in functional disability have been major components of successful aging and have become key public health priorities in many aging societies, including Taiwan[1]. In recent decades, researchers have paid increasing attention to how leisure time activity (LTA), which is one of the major components of a healthy lifestyle with an emphasis on active engagement with life, prevents functional disabilities [2–4]. Studies also have shown that engagement in LTA in later life, and increases in LTA engagement, is associated with a slower speed of progression toward functional disability [5, 6].

Evidence has shown that sociodemographic factors such as older age, female gender, lower educational attainment, lower self-rated health, and a greater number of comorbidities and depressive symptoms are commonly found as predictors of higher functional disabilities [7–11] and of LTA participation [12]. Liang (2010) has emphasized the importance of examining the role of health behaviors (such as participation in LTA) as mediating factors between older adults’ sociodemographic factors and their physical function. However, there are no studies investigating how much LTA may offset the influence of demographic factors on older adults’ functional disabilities [7]. Consequently, the goal of this study was to gain insight into both the mechanisms through which sociodemographic factors are associated with older adults’ development of functional disability and the role played by LTA participation in mediating the relationship between sociodemographic factors and functional disability.

Levels of LTA and levels of functional disability both change over time, especially for older adults, who may suffer from newly acquired diseases or the general effects of aging. We applied latent growth curve modeling (LGCM) to examine the LTA process as a mediational process between older adults’ sociodemographic factors and the outcome process of functional disabilities [13, 14]. The aims of this study were to investigate (1) whether and (2) the extent to which LTA trajectories mediated the potential associations between six common sociodemographic factors and functional disability trajectories among Taiwanese older adults.

## Materials and methods

The current study has been approved by the Research Ethics Committee of National Taiwan University (2013HS064).

### Data and sample

The Taiwan Longitudinal Study on Aging (TLSA) was launched in 1989 and was followed up in 1993, 1996, 1999, 2003, and 2007 by the Taiwan Provincial Institute of Family Planning and the University of Michigan, with support from Taiwan’s government and the U.S. National Institute on Aging. The data from the TLSA have been used in numerous health-related analyses due to its survey design and quality [7, 15]. The survey used a complex sampling survey design with aged-in cohorts to represent the distribution of both community- and institution-dwelling older adults in Taiwan. The first cohort, in the 1989 survey, was a sample of 4,049 adults aged 60 and older who were interviewed face to face. A second cohort aged 50 to 66 was added in the 1996 survey, which included 2,462 older adults. The response rate for all waves of the survey was very high, ranging from 98.1% in the 1996 survey to 95.7% in the 2007 survey [15].

Some key variables and covariates are available only from the data collected in the 1996 survey and the three following surveys. Thus, only four waves of survey data—from the 1996, 1999, 2003, and 2007 surveys—were included in this study for analysis. This study included 3,429 older adults who survived to the 2007 survey and had completed at least one of the four surveys between 1996 and 2007. Sample weights were included to ensure representativeness of Taiwan’s population aged 50 and older as of 1996. Missing values were replaced using the Markov Chain Monte Carlo method through the multiple imputation procedure in Mplus 7.3 with 10 data sets imputed [16].

## Measures

### Sociodemographic factors as predictors

The current study selected six commonly studied sociodemographic factors considered to be predictors of older adults’ disability and LTA participation: age, gender, education level, self-rated health, number of comorbidities, and depression. The data for these six factors were extracted from the baseline TLSA survey (the 1996 survey). The number of comorbidities was the number of reported chronic health conditions (e.g., hypertension, diabetes mellitus, heart disease, stroke, cancer, pulmonary disease, arthritis, gastric ulcer, liver disease, hip fracture, cataract, renal disease, gout, spinal spurs). Self-rated health was assessed with this question: “Regarding your state of health, do you feel it is excellent [coded 5], good, average, not so good, or very poor [coded 1].” Depression was assessed by the 10-item version of the CES-D, which measures levels of depressive symptoms on a scale ranging from 0 to 30 [17].

The study participants were 50.02% female, with a mean age of 63.92 (SD = 8.26) years and an average of 5.08 (SD = 4.66) years of education and 1.37 comorbidities (SD = 1.52) in 1996. The average score of self-rated health was 3.34 (SD = 1.08) in 1996.

### Leisure time activities as mediating variables

Both physically active and sedentary types of LTA were included in the current study, with a total of eight activities. Older adults were asked, “Do you usually engage in any kind of leisure time activity?” and were given the following types of activities to choose from: (1) listening to music or the radio, (2) reading, (3) playing mah-jongg or chess, (4) gathering with friends or family, (5) gardening, (6) taking a walk, (7) participating in outdoor activities, and (8) participating in group activities. The frequency of each activity was assessed using five levels, from *none* (0) to *about every day* (4).

In our preliminary analysis, LTA fit better as one composite (LTA: χ^2^ [2, *N* = 3,429] = 102.51, *p* < .001; CFI = .968; RMSEA = .121; SRMR = .026) rather than as the two composites of physically active LTA and sedentary LTA (χ^2^ [19, *N* = 3,429] = 540.10, *p* < .001; CFI = .916; RMSEA = .089; SRMR = .039). We therefore included LTA in the final analysis as a composite score, with a range from 0 to 32. A higher score indicated higher participation in LTA. The mean LTA participation score was 9.61 (*SD* = 5.84) in 1996; it then increased to 10.27 (*SD* = 5.78) and 9.54 (*SD* = 5.59) in 1999 and 2003 respectively, and then decreased to 8.99 (*SD* = 5.74) in 2007 (Table 1).

**Table 1:** Characteristics of the sample responding in each wave (*N* = 3,429).

	Interviewed year			
Sample characteristics	1996	1999	2003	2007
Age	63.92(8.26)	66.92(8.26)	70.25(8.25)	74.31(8.25)
Female	1711(49.90)	1711(49.90)	1711(49.90)	1711(49.90)
Education years	5.08(4.66)	5.08(4.66)	5.08(4.66)	5.08(4.66)
Self-Rated Health	3.33 (1.062)	3.27(1.023)	3.12(1.052)	2.93(0.998)
Comorbidities	1.39(1.501)	1.39(1.421)	1.83(1.642)	2.08(1.692)
Depression	5.09(5.341)	4.72(5.217)	5.17(5.482)	5.76(5.867)
Leisure time activities (LTA, 0–32)	9.61(5.84)	10.27(5.78)	9.54(5.59)	8.99(5.74)
Functional limitation (FL, 0–24)	1.50(3.25)	2.47(4.13)	3.91(5.41)	5.50(6.78)
Instruction activity of daily living (ADL, 0–18)	0.81(2.26)	1.11(2.69)	2.05(3.99)	3.52(5.44)
Activity of Daily Living (ADL, 0–18)	0.10(0.97)	0.18(1.20)	0.53(2.34)	1.55(4.22)

### General functional disabilities as outcome variables

The current study applied multiple-indicator LGCM, with a latent variable for general functional disability (GFD) measured at four time points: 1996, 1999, 2003, and 2007. Three indicators—activities of daily living [18], instrumental activities of daily living [19], and Nagi’s functional limitations [20]—were used to assess the latent variables of GFD at each time point.

The National Research Council has suggested that including functional limitations in addition to ADL and IADL limitations allows researchers to better understand the GFD process[21]. These three indicators assess older adults’ physical function from multiple perspectives, from light to heavy disability. The four latent variables serve to explain variance in and covariance among these three indicators at different time points. Multiple indicators in a growth model provide researchers with greater flexibility in modeling complex patterns of change and greater power to detect individual differences in change [5, 22–24].

All interviews for the four waves of the TLSA data used in this study included identical sets of questions for self-reported information on disability status and physical performance. For Nagi’s functional limitations, respondents were asked about any difficulty experienced with eight physical functions, such as grasping or raising hands overhead, without outside help or the use of aids. For IADLs, respondents were asked whether they had any difficulty conducting six activities, such as shopping or managing money, by themselves. For ADLs, respondents were asked about any difficulty not of a temporary nature in carrying out six activities, such as bathing or dressing independently (see Table 1). The severity level for each of the activity of the three indicators—Nagi’s functional limitation, ADL disability, and IADL disability—was assessed with four grades, from 0 (*no limitation*) to 3 (*unable to do*). The severity level for each activity was summed up separately. As a result, the ranges for severity of functional limitation, IADL disability, and ADL disability were 0 to 24, 0 to 18, and 0 to 18, respectively, at each time point. Higher scores indicated greater limitations in physical performance.

Study participants started out with less severe disabilities in 1996, with 1.50 (*SD* = 3.25) for Nagi’s functional limitations, 0.81 (*SD* = 2.26) for IADLs, and 0.10 (*SD* = 0.97) for ADLs. Functional disabilities continually increased over the years, with more severe disability measured in Nagi’s functional limitations (5.50; *SD* = 6.78), IADLs (3.52; *SD* = 5.44), and ADLs (1.55; *SD* = 4.22) in 2007.

### Analysis

To examine the longitudinal relations among the study variables, parallel-process LGCM was conducted [14, 25], which allowed us to examine the growth trajectories of GFD and LTA simultaneously [25]. In the past few decades, parallel-process LGCM has been recommended to evaluate the mediational process in longitudinal relationships, especially when both mediator and outcome variables change over time [14]. The growth of the mediator variables and the growth of the outcome variables can be viewed as two distinct processes. In the future, the mediational process can be viewed as a prevention program that influences the growth of the outcome indirectly [14].

**Fig 1:**
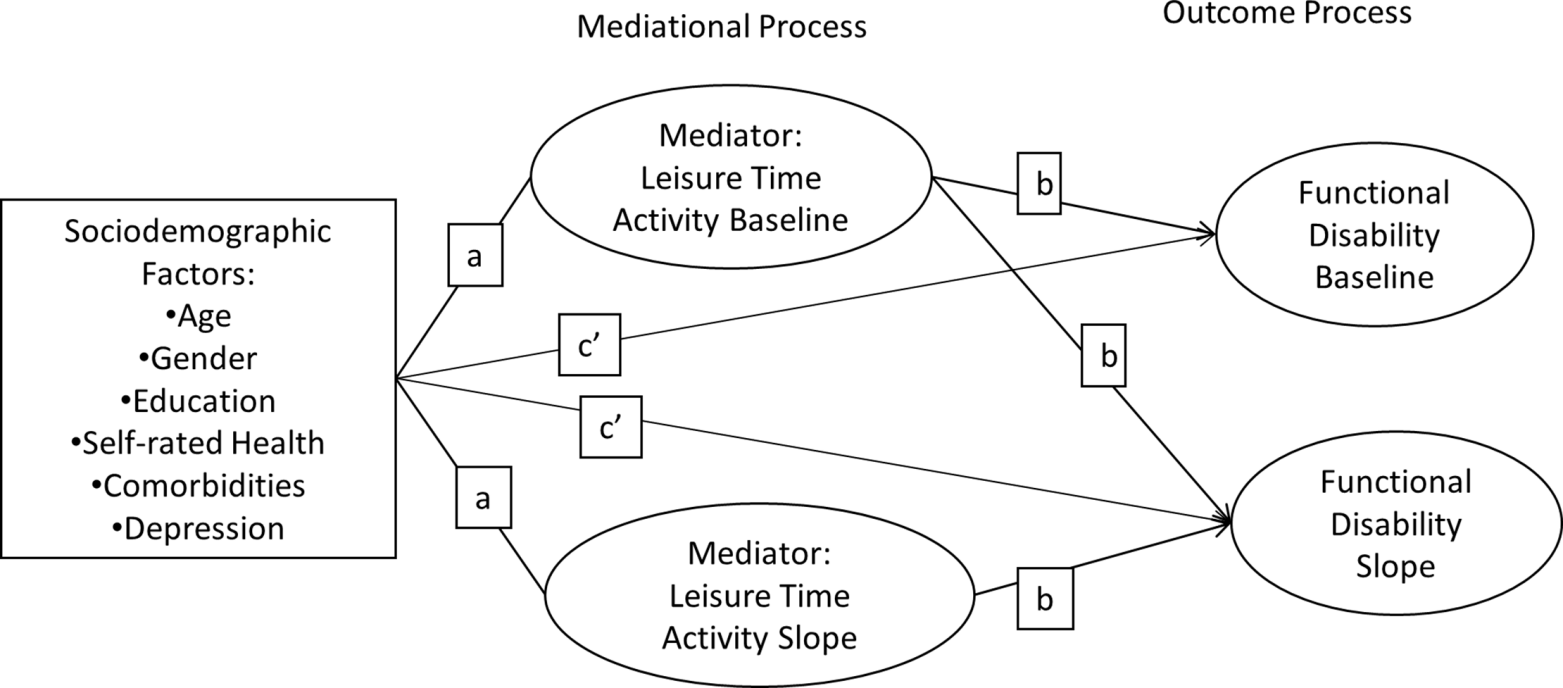
Mediation model with leisure time activity as mediator between six predisposing factors and functional disability.

This study followed the steps suggested by Muthen and Cheong, Mackinnon for testing the mediational process in parallel-process LGCM[14, 26]: First, the growth trajectory shapes of the LTA process and the GFD process were investigated individually. In this step, the two processes (measurement models for LTA and GFD) were to analyze the patterns of change in LTA and GFD and to test for linear (0, 3, 7, 11), quadratic (0, 9, 49, 121), or unspecified curve (0, freely estimated, freely estimated, 11) trajectories. We first tested measurements of GFD for invariance across time before pursuing further model testing [27, 28]. The measurement errors were set to be correlated [29]. Second, the two processes investigated in the first step were combined into one parallel-process model. Finally, the six selected predisposing factors were introduced to the parallel-process model tested in Step 2 (please see Fig 1). The effects of the sociodemographic factors on the two mediating latent variables (LTA baseline and LTA slope) and on the two outcome latent variables (GFD baseline and GFD slope) were represented as a-path and c'-path, and the effects of the two mediating latent variables on the two outcome variables were represented by b-path.

In the current study, we hypothesized (1) six mediating effects from the six predictors to the baseline GFD through the baseline LTA; (2) six mediating effects from the six predictors to the rate of change in GFD through the baseline LTA; and (3) six mediating effects from the six predictors to the rate of change in GFD through the rate of change in LTA.

The estimates of the mediated effect (ab), direct effects (c'), and total effect (ab+c') of the baseline value of and rate of change in LTA were tested for significance [14, 26, 30, 31]. The total effects were calculated and tested based on outcome latent variables, which summarized all effects of the sociodemographic factors on the GFD baseline and on the GFD slope respectively. A significant *p* value was set as less than .05. The proportion mediated was used to evaluate the effect size of mediation following this formula: ab/(ab+c') [13]. If the mediated effect or the total effect was not statistically significant, the proportion mediated was not calculated [13].

Mplus (version 7.3) was used to estimate all mediated effects with a robust maximum likelihood estimator. The following goodness of fit indices were used: (1) chi-square statistics [32], (2) the Bentler Comparative Fit Index (i.e., CFI ≥ 0.9;[33, 34], (3) root mean square error of approximation (i.e., RMSEA ≤ 0.05) with a 90% confidence interval (CI; [35], and (4) standardized root mean square residual (i.e., SRMR ≤ 0.08; [32, 36]. Sample weights were included for analysis [37].

## Results

### Patterns of LTA and GFD

Table 1 shows LTA and GFD data from 1996 to 2007 for the total sample in different gender and age groups. Male older adults were more frequently active than female older adults (*p* < 0.05). Level of GFD increased across the period (*p* <0.001). Female older adults tended to have higher level of GFD. As participants get older, they tended to decrease their LTA participation (*p* < 0.01) and their disabilities increased as well (*p* < 0.01).

### Measurement model

Both the functional disability process (χ^2^ [44, *N* = 3,429] = 431.77, *p* < .001; CFI = .960; RMSEA = .051; SRMR = .081) and the LTA process (χ^2^ [5, *N* = 3,429] = 85.08, *p* < .001; CFI = .974; RMSEA = .068; SRMR = .029) tested in Step 1 fitted well to the data. The GFD process holds factor loading invariance and partial intercept invariance across time. The parallel-process model tested in Step 2 also showed good fit to the data (χ2 [164, N = 3,429] = 1,838.25, *p* < .001; CFI = .931; RMSEA = .055; SRMR = .061) (see S1 Table for information on measurement models.) Thus, we proceeded to the final step for mediational process testing.

### Mediational model

Our main interest in this study was whether the LTA trajectory served as a mediating process that changed the relationships between the six predictors and the GFD process. The mediational process model fitted the data well (χ2 [164, N = 3,429] = 1,838.25, *p* < .001; CFI = .931; RMSEA = .055; SRMR = .061). The estimates of these relationships are shown in Table 2. Among the 18 mediating effects tested in the study, 15 were significant. Of all the predictors, only education and comorbidities showed no direct impact on the rate of change in GFD; the effect of education and comorbidities were totally mediated through the baseline LTA and the rate of change in LTA. Among the 15 significant indirect paths, six showed inconsistent mediation, indicating that the direct and indirect effects are opposite. Detailed results on the mediating effects are reported in Table 2.

**Table 2:** Summary of the mediating effects of leisure time activity on general functional disability (N = 3,429).

Mediational Model							
Paths	Mediators (ab)		Direct Effect (c')		Total Effect (ab + c')		Proportion Mediated ab/(ab +c')
		Estimates	Standardized Estimates	Estimates	Standardized Estimates	Estimates	Standardized Estimates	Ratio
Exogenous Variables → Baseline LTA → Baseline GFD							
Age	-0.009***	-0.034***	0.060***	0.218***	0.051***	0.183***	0.186
Gender	0.089***	0.021***	0.365***	0.087***	0.454***	0.108***	0.194
Education	-0.055***	-0.120***	0.026*	0.058*	-0.028***	-0.062***	1.935
Self-Rated Health	-0.069***	-0.035***	-0.323***	-0.164***	-0.391***	-0.199***	0.176
Comorbidities	-0.026**	-0.018**	0.120***	0.084***	0.094*	0.066**	0.273
Depression	0.012***	0.030***	0.069***	0.172***	0.081***	0.202***	0.149
Exogenous Variables → Baseline LTA → Slope of GFD							
Age	-0.002***	-0.041***	0.011***	0.209***	0.022***	0.417***	0.098
Gender	0.020***	0.026***	0.088***	0.110***	0.077***	0.096***	0.271
Education	-0.013***	-0.144***	-0.005	-0.056	-0.008***	-0.095***	1.516
Self-Rated Health	-0.016***	-0.042***	-0.001	-0.002	-0.012	-0.032	-
Comorbidities	-0.006**	-0.022**	0.014	0.050	0.031***	0.114***	0.125
Depression	0.003***	0.036***	-0.002	-0.031	-0.001	-0.008	-
Exogenous Variables → Slope LTA → Slope of GFD							
Age	0.013***	0.245***	0.011***	0.209***	0.022***	0.417***	0.590
Gender	-0.034	-0.042	0.088***	0.110***	0.077***	0.096***	0.438
Education	0.009***	0.106***	-0.005	-0.056	-0.008***	-0.095***	1.116
Self-Rated Health	0.006	0.016	-0.001	-0.002	-0.012	-0.032	-
Comorbidities	0.023***	0.084***	0.014	0.050	0.031***	0.114***	0.737
Depression	-0.001	-0.018	-0.002	-0.031	-0.001	-0.008	-
χ2 [164, N = 3,429] = 1,838.25, *p* < .001; CFI = .931; RMSEA = .055; SRMR = .061							

Note

* *p* < .05

** *p* < .01

*** *p* < .001.

### Mediational model: Six predictors to baseline GFD through baseline LTA

For hypothesis 1, Table 2 shows that for all six predictors, baseline LTA served as a significant mediator toward a lower baseline GFD. These findings imply highly significant mediational processes in which age (*β* = -0.034, *p* < .001), education (*β* = -0.120, *p* < .001), self-rated health (*β* = -0.035, *p* < .001), and comorbidities (*β* = -0.026, *p* < .001) were negatively associated with baseline GFD through baseline LTA participation. Thus older adults with greater age, education, self-rated health, and comorbidities were more likely to benefit from baseline LTA participation, in the form of a lower baseline GFD.

It is important to note the inconsistent mediating effects of baseline LTA participation on the relationships between age, education, number of comorbidities, and baseline GFD. On their own, each of these three sociodemographic factors was found to be a risk factor for GFD outcomes, yet their indirect effects when mediated through LTA participation instead protected older adults from developing GFD. For example, the standardized mediating effect of education on a lower baseline GFD through baseline LTA had almost 2 times the magnitude of its direct effect on baseline GFD, and in the opposite direction. Meanwhile, education had 3 to 6 times the effect on GFD outcomes as the other sociodemographic predictors. The proportion of the total effects on baseline GFD that was mediated through baseline LTA participation was 194% for education, 18.6% for age, 17.6% for self-rated health, and 14.9% for depression.

### Mediational model: Six predictors to change in GFD through baseline LTA and slope of LTA

For hypotheses 2 and 3, Table 2 shows that for all six predictors, the baseline LTA served as a significant mediator toward a slower increase in GFD over time. These findings imply highly significant mediational processes in which age (*β*_*Through baseline LTA*_ = -0.041, *p* < .001), education (*β*_*Through baseline LTA*_ = -0.144, *p* < .001), self-rated health (*β*_*Through baseline LTA*_ = -0.042, *p* < .001), and number of comorbidities (*β*_*Through baseline LTA*_ = -0.022, *p* < .001) were negatively associated with changes in GFD through baseline LTA participation. As each of these demographic factors increased, older adults became more likely to receive benefits from LTA participation, in the form of a slower rate of increase in GFD.

The rate of change in LTA also served as a significant mediator to the rate of change in GFD, but for only three predictors: age (*β*_*Through slope of LTA*_ = 0.245, *p* < .001), education (*β*_*Through slope of LTA*_ = 0.106, *p* < .001), and number of comorbidities (*β*_*Through slope of LTA*_ = 0.084, *p* < .001). This suggests that as age, education level, or number of comorbidities increase, older adults are less likely to increase their rate of LTA participation, and that they may therefore indirectly experience a higher risk of faster increase in GFD. However, the effects were minor.

The inconsistent mediating effects of baseline LTA participation on the relationships between age, education, number of comorbidities, and change in GFD were strong. The direct effects showed that as age, education, and number of comorbidities increased, older adults were at higher risk of developing disability at a faster rate. However, we found that the indirect effects of these three predictors, mediated through baseline LTA participation, protected older adults who had become disabled from a faster rate of progression toward GFD.

Among these three predictors, education and comorbidities even showed a total mediating effect toward slower rate of change in GFD, even given the small but positive mediating effect of education and comorbidities toward faster progression of disability through the rate of change in LTA. This indicates that there were no direct impacts of education level and number of comorbidities on changes in older adults’ GFD, although there were significant indirect effects mediated through the baseline and rate of change in LTA participation. It further indicates that as older adults’ education level increases and number of comorbidities, LTA participation is required for them to benefit from a slower speed of progression toward GFD. The proportions of the mediation effect of education and number of comorbidities on the rate of change in GFD through the baseline LTA were 151.6% and 12.5% respectively, and through the rate of change in LTA participation were 111.6% and 73.7% respectively.

## Discussion

A key contribution of this research lies in its focus on the effects of the LTA trajectory as a mediator between sociodemographic factors and GFD trajectory among older adults. For all six sociodemographic factors, both baseline LTA and change in LTA demonstrated either partial or total mediation effects on GFD trajectories. The proportion of effects mediated by the LTA trajectory ranged from 9.8% (for age to change in GFD) to 194% (for education to baseline GFD). Our findings provide evidence that LTA plays an important role in mediating the relationship between sociodemographic factors and late-life functional disabilities. These findings underscore the importance of addressing public health policy and developing LTA related health programs for older adults.

### Education can be a protective factor, only if older adults engage with LTA participation

A higher education level has long been shown to be a protective factor toward later onset or lower risk of developing functional disability in later life [10, 38]. Martin, Zimmer (2011) suggested that older adults with more education may invest in late-life health through healthier behavior, and hence be at less risk of developing functional disabilities. The current study provides strong empirical evidence for the assumption that the protective effect of education is strongly and even fully mediated through LTA participation. This advances our knowledge regarding the influence of education on the development of functional disabilities in older adults[10].

It is interesting to notice that when LTA participation was considered, education itself was shown to be a weak risk factor toward developing functional disabilities. Thus, in addition to providing empirical evidence of the mediating role between education and physical function, the current study findings further suggest that education may be a better protective factor for those older adults who participate in LTA. Even for older adults who have developed disabilities, a higher education may lead to increased LTA participation and therefore result in a slower progression toward disability. Bengtsson and Datta Gupta (2017) suggested that education has a potential causal effect on disabled people’s ability to cope with disabilities. Our findings might shed some further light on the relationship in that older adults with a higher education level may participate more in LTA, and that greater level of LTA participation may therefore slow the progression toward disability and lead to better adjustment[39].

In summary, education, through LTA participation as a mediator, can have strong effects on older adults having a lower baseline and a slower rate of progression of disability. The effects of education were as much as 3 to 6 times the size of the effect of the other sociodemographic factors. The large proportion of effects mediated through baseline LTA further emphasizes the importance of LTA as a mediator in this relationship. Promoting older adults’ awareness of the benefit of LTA participation may be a promising strategy for preventing functional disabilities.

It is worth noting that as education level increases, older adults were less likely to increase their LTA participation over time, and they therefore suffered a minor setback in the related benefits. Past studies have pointed out that compared to baseline levels of LTA, increasing the rate of change in LTA has more than 3 times the effect on slowing down the progression of disabilities [5]. Thus, as older adults’ education level increases, encouragement to increase their level of LTA participation or to be involved in a wider range of types of LTA could lead to further benefits in slowing the rate of progression toward disability.

### LTA participation may promote successful aging

Like education, the factors of age and number of comorbidities also showed inconsistent mediation effects. These two factors were risk factors for GFD development, but the indirect effects mediated through LTA participation were found to protect older adults from developing GFD. Although the negative effects of age and comorbidities and the positive effects of engaging in LTA on older adults’ functional disabilities have been well studied separately in the literature [3, 7, 10, 11, 40, 41], this may be the first study to pull these three factors together to show the mechanism by which older adults may benefit from LTA participation as they age and develop comorbidities.

A large proportion of the mediating effects of baseline LTA participation were found to be inconsistent, which is an exciting finding. LTA participation may offset the degree and rate of increase in disability that accompanies the aging process and may also offset the impact of comorbidities. Through good baseline LTA participation, older adults may benefit from a lower baseline GFD (18.6% of total effects mediated) and from a slower speed of increase in GFD (9.8% of total effects mediated) as their age increases. Older adults with comorbidities may benefit from baseline LTA participation with a lower baseline GFD (27.3% of total effects mediated) as well as a slower speed of increase in GFD (19.3% of total effects mediated). Maintenance of physical function and active engagement with life are key to aging successfully [1]. This study’s findings support the idea that LTA participation may be a way to age successfully [3].

### Male and female older adults may have different attitudes toward LTA

Past research findings have pointed out that older women bear a disproportionately large burden of disability compared to older men: older women are more functionally impaired than older men and also experience greater increases in disability over time [7, 15]. Chen further suggested that, when considering disability in trajectories, older women bear a higher risk of faster rates of increase in GFD once it has developed[24].

Our study’s findings show that this gendered disparity might be due to different levels or types of LTA participation among male and female older adults. Even after adjusting for education, baseline LTA participation still showed significant direct and indirect effects between gender and the disability trajectory. Strobl and Muller reported that while men benefited from midlife physical LTA with respect to the development of late-life disability, the same effect was not observed in women, but the researchers did not describe a mechanism that might cause such differences[42]. Our findings suggest that older women are less likely than their male counterparts to build a high level of baseline LTA and continue to increase their participation in LTA, which indirectly results in a higher baseline GFD and a faster increase in GFD. However, past study findings related to gender differences in LTA participation have not been consistent: some studies reported no gender differences in LTA participation [43], while others reported that women were more likely than men to continue their participation in social activities into later life [12] but were less likely to participate in all types of physically active LTA [44]. The proportion of the effects (ranged from 19.4% to 27.1%) of gender that were mediated by LTA participation indicates that further investigation is merited, including into the extent to which gender differences in outcomes are due to differing characteristics of or differing levels of LTA participation among older men and older women.

### Self-rated health influences GFD only through concurrent status of LTA

A high level self-rated health has been well studied in the literature as a protective factor against functional disability in older adults [45, 46] and as a positive effect of continued participation in LTA [12, 44]. While the current study echoes findings from past studies, it also adds to the literature a mechanism by which LTA participation may reinforce the relationship between self-rated health and functional disability in late life.

The proportion of the effects of self-rated health that were mediated by LTA participation suggests the importance of promoting LTA for people who rate themselves healthy. Without such encouragement, some may achieve less benefit than they could, by reducing their activity levels after rating themselves healthy. It was interesting to note that among the surveyed older adults, higher self-rated health did not have any direct or indirect effect on change in GFD, which may indicate that self-rated health influences baseline and changes in GFD only through concurrent LTA participation. Increases in self-rated health had no impact on later change in LTA participation and therefore no impact on change in GFD. Strain and Grabusic (2002) reported that better or same-as-previous self-rated health reports over an 8-year study period were associated with continued activities such as walking or outdoor activities; the study did not examine changes in the level of these activities[12]. The evidence from the current study and the past literature also shows no effect of self-rated health on the future change in GFD when mediated through change in LTA. Further study is recommended to examine the role of the LTA trajectory or just the baseline LTA as mediators for self-rated health and GFD trajectory in late life.

### Older adults with depression may not benefit from LTA

LTA participation showed no beneficial mediating effects on the relationship between older adults’ depression scores and their baseline level of and rate of growth in GFD over time. This was a surprise to our research team. Many studies have suggested a strong association between depression and older adults’ development of functional disabilities [8, 46, 47], including that LTA may decrease older adults’ depressive symptoms [4, 48–50]. Our findings, however, suggest that LTA participation does not buffer this relationship.

It is likely that older adults with depression are less likely to engage in LTA; this choice therefore indirectly leads to a higher baseline GFD and a faster increase in GFD. Ku and colleagues [51] found that engagement in physically active LTA in later life was associated with a lower risk of subsequent depressive symptoms, but the reverse association is not supported. Ku and colleague' findings might partially explain our study’s findings regarding why LTA participation did not serve as a mediators between older adults' depression symptom and GFD. In summary, encouraging older adults with symptoms of depression to engage in LTA may have a very limited effect on disability prevention or maintenance. To better care for older adults with depressive symptoms, further examination of factors that might provide a buffer against depression and disabilities is merited.

### Increased level and types of LTA participation may be crucial to successful aging

It is important to note that the rate of growth in LTA was a mediator for three predictors, which have shown indirect effects pointing toward a faster increase of GFD. The older adults become older, the higher the education level they’ve achieved, or the more comorbidities they have, the less likely they are to participate in LTA [12], and this may indirectly lead to a faster increase in GFD once it occurs. However, a high proportion of these indirect effects were mediated by LTA participation—ranging from 59% for age to 111% for education—which suggests that greater than 59% of the effects of the sociodemographic factors on the faster increase in functional disabilities could be modified by health behavior.

Past research studies have explored the factors and health behaviors that contribute to a higher risk of developing functional disabilities in late life. The current study is the first to investigate the extent to which health behavior may play a mediating role in this relationship. Encouraging older adults to explore their interests and participate in LTA or, if already engaging in LTA, to increase their LTA participation, may be an important strategy for maintaining health in future aging societies. Past research studies have encouraged older adults to participate in more varied types of LTA for better mental health and quality of life [50]. Our study’s findings add that engagement in a wider variety of LTA is crucial for older adults' GFD development, and especially for those with specific sociodemographic characteristics, to experience a slower progression in functional disability and lead to successful aging. Findings from the current study also provide information on which populations of older adults should be priorities for encouraging participation in a wider variety of LTA.

### Limitations

The strengths of this study are the large sample size, the long follow-up period, the small loss to follow-up, and, most importantly, the use of parallel-process LGCM with specific information on the baseline level of and change in LTA and GFD. However, some limitations need to be addressed. For the parsimony of the model, we investigated only six of the commonly studied predictors considered to be determinants of GFD. Future studies could investigate whether older adults’ LTA can modify the relationship between other predisposing factors and functional disability trajectories.

The older adults included in the current study were those who completed the 1996 TLSA survey and survived to 2007. To investigate how older adults’ LTA trajectories may mediate between predictive factors and GFD trajectories, it was necessary to include surviving older adults. However, when compared with the overall older population in the survey, this study’s sample may be biased toward younger age and greater proportion female. Our results may only represent this specific set of older individuals who survived the 11-year period from 1996 to 2007 in Taiwan (please see S2 Table for details), and may be generalizable only to those who are less disabled.

Another potential limitation is that the current study is based on a large Taiwanese data set. Older adults in Taiwan, compared to those in Western countries, may view LTA differently [52]. Thus, findings from the current study may be generalizable only to societies with similar cultures.

As we have pointed out, prior studies of the influence of LTA on older adults’ functional disability trends, especially ones that studied older adults in Asia, have been scarce [2, 43, 53, 54]. Findings from the current study could add valuable information on how LTA is related to functional disability trajectories in Asian populations. However, some of the findings may have less association with cultural differences and may still be of great value to older adults from other cultures.

## Conclusion

This study clearly shows that LTA may serve as a potential positive intervention in the relationship between older adults’ sociodemographic characteristics and development and progression in functional disability. The relationships between functional disability and age, education, and number of comorbidities—three of the six sociodemographic factors considered here—may in particular be positively affected by strong LTA participation, given the high proportion of effects mediated. Our study findings provide practical suggestions for better targeting of subpopulations of older adults to promote healthy behaviors. LTA is key to helping older adults maintain an active lifestyle and may be particularly important for successful aging in Asian countries, where intensive physical exercise is less common than in Western countries.

## Supporting information

S1 Table Results of measurement models for general functional disability and leisure time activity. (DOCX)

S2 Table Baseline characteristics (in 1996) of the total population and the sample included and excluded from the study. (DOCX)
