# Supplementary material for: Leisure time activities as mediating variables in functional disability progression: An application of parallel latent growth curve modeling
Source: PLoS One. 2018 Oct 3;13(10):e0203757. doi: 10.1371/journal.pone.0203757 (PMC6169861; doi:10.1371/journal.pone.0203757)
Supplement: S1 Table — (DOCX) [file pone.0203757.s001.docx]

Supplementary Table 1 Results of Measurement Models for General Functional Disability and Leisure Time Activity (N = 3,429)

|  | Measurement Models | | | | | |
| --- | --- | --- | --- | --- | --- | --- |
| Goodness of Fit Indices | GFD LGCM | | | LTA LGCM | | |
| χ2 [df], *p* value | 431.77[44], <0.001 | | | 85.08[5], <0.001 | | |
| CFI | 0.960 | | | 0.974 | | |
| RMSEA | 0.051 | | | 0.068 | | |
| SRMR | 0.081 | | | 0.029 | | |
| Intercept | 0.000 | | | 9.846 | | |
| Slope | 0.115 | | | -0.028 | | |
| Coefficients |  | | |  | | |
| Slope | GFD Estimates | GFD SE | GFD Residuals | LTA Estimates | LTA SE | LTA Residuals |
| on 1996 data | 0.000 | 0.000 | 1.001 | 0.000 | 0.000 | 16.156 |
| on 1999 data | 2.449 | 0.366 | 2.237 | 3.000 | 0.000 | 16.559 |
| on 2003 data | 9.423 | 0.584 | 1.931 | 7.000 | 0.000 | 13.789 |
| on 2007 data | 11.000 | 0.000 | 15.324 | 11.000 | 0.000 | 11.141 |
| The parallel-process model: χ2 [164, N = 3,429] = 1,838.25, *p* < .001; CFI = .931; RMSEA = .055; SRMR = .061 | | | | | | |

Note: Only unstandardized coefficients, standard errors, and residuals are included. FLxxxx ^a^ = Nagi’s functional limitation in xxxx (year); ADLxxxx^b^ = activities of daily living in xxxx (year); IADLxxxx^c^ = instrumental activities of daily living in xxxx (year); GFDxxxx^d^ = general functional disability in xxxx (year).

* *p* < .05; ** *p* < .01; *** *p* < .001.
