# Supplementary material for: Leisure time activities as mediating variables in functional disability progression: An application of parallel latent growth curve modeling
Source: PLoS One. 2018 Oct 3;13(10):e0203757. doi: 10.1371/journal.pone.0203757 (PMC6169861; doi:10.1371/journal.pone.0203757)
Supplement: S2 Table — (DOCX) [file pone.0203757.s002.docx]

Supplementary Table 2. Baseline Characteristics (in 1996) of the Total Population and the Sample Included and Excluded from the Study (N = 3,429)

| Characteristics | Total population | Subgroups of population | | |  | Difference test |
| --- | --- | --- | --- | --- | --- | --- |
|  |  | Included | Excluded (deceased) | Excluded  (lost to follow-up) |  |  |
| Number of cases (%)^a^ | 5451(100) | 3429(62.91) | 1981(36.34) | 41 (0.75) |  | -- |
| Age (*SD*)^a^ | 67.16 (9.38) | 63.92 (8.26) | 72.59 (8.59) | 75.90 (6.88) |  | * |
| Female (%)^a^ | 2507 (45.99) | 1711(49.90) | 781 (39.42) | 15 (36.59) |  | * |
| Years of education (*SD*) ^a^ | 4.64 (4.61) | 5.08 (4.66) | 3.86 (4.39) | 5.34 (5.17) |  | * |

Note:

1. Statistics are shown as mean (*SD*) for continuous and frequency (%) for categorical variables. The characteristics are examined at the baseline year (1996).

**p < .05;* ** *p < .01;* *** *p < .001.* The difference test among the samples is examined by one-way ANOVA or chi-squared test.
